# Exploring cross-modal plasticity in the auditory–visual cortex post cochlear implantation: implications for auditory and speech function recovery and mechanisms

**DOI:** 10.3389/fnins.2024.1411058

**Published:** 2024-08-19

**Authors:** Xiao-Feng Qiao, Lu-Dan Liu, Ling-Yan Han, Ying Chen, Xin Li

**Affiliations:** ^1^Department of Otorhinolaryngology, The Fifth Clinical Medical College of Shanxi Medical University, Taiyuan, China; ^2^Graduate School, Shanxi Medical University, Taiyuan, China; ^3^Department of Surgery, Children's Hospital of Shanxi Province, Taiyuan, China

**Keywords:** auditory function, auditory–visual cortical activity, cochlear implantation, cross-modal functional reorganization, speech function

## Abstract

**Objective:**

The aim of this is to explore changes in cross-modal reorganization within the auditory–visual cortex after cochlear implantation, examining their influence on auditory and speech functions along with their underlying mechanisms.

**Methods:**

Twenty prelingually deaf children who received cochlear implantation and rehabilitation training at our hospital between February 2022 and February 2023 comprised the prelingual deaf group. Simultaneously, 20 healthy children served as the control group. The prelingual deaf group underwent brain cortical activity assessment and evaluation of auditory-speech recovery pre-surgery, at postoperative weeks 1 and 2, and at months 1, 3, 6, 9, and 12. The control group underwent parallel assessments and evaluations. We analyzed the correlation between cortical activity in the auditory–visual cortex of patients and their auditory-speech functional recovery.

**Results:**

The group with prelingual deafness displayed elevated levels of auditory and visual cortical electromagnetic intensity compared to the control group, both prior to and 9 months after surgery. However, by the 12-month mark post-surgery, there was no discernible distinction between the two groups. Following surgery, the prelingually deaf group exhibited a progressive improvement in both Categories of Auditory Performance (CAP) and Speech Intelligibility Rate (SIR), initially lagging behind the control group. Notably, a negative correlation emerged between auditory and visual cortical electromagnetic intensity values and CAP/SIR scores at the 12-month post-surgery assessment.

**Conclusion:**

Cochlear implantation in prelingually deaf children results in elevated activity within the auditory and visual cortices, demonstrated by heightened electromagnetic intensity readings. Cross-modal reorganization is observed temporarily at 3 months post-surgery, which resolves to baseline levels by 12 months post-surgery. This phenomenon of reversal correlates with the restoration of auditory and speech functions in these children.

## 1 Introduction

Cochlear implant is a common way to treat deafness, which can replace the inner ear hair cells in the diseased area and cause direct electrical stimulation to the auditory nerve fibers to promote the production of hearing (Saki et al., 2022). Although cochlear implants can help hearing-impaired children recover hearing and speech function, the effect after implantation still varies greatly (Niparko et al., 2010; Geers et al., 2011), especially in noisy environments (Saksida et al., 2022). It is not clear why some children with cochlear implants have poor audiotory and speech perception after implantation. Many factors, such as strategy for rehabilitation of communication, age of onset of hearing loss, duration of deafness, age of cochlear implantation, experience of wearing hearing-Aids, and duration of use of cochlear implantation, have an impact on the outcome of auditory and speech perception, but the huge differences in the development of auditory skills in children with cochlear implantation remain unexplained (Zeng, 2004; Tomblin et al., 2005; Lin et al., 2008; Niparko et al., 2010; Tobey et al., 2013). In recent years, studies have found that after the loss of hearing function, the brain will use the auditory cortex to assist in processing when the visual cortex processes stimulus signals. Visual compensation causes changes in the cerebral cortex, which is clinically called brain cross-modal functional recombination (Stroh et al., 2022). This neuroplasticity may provide adaptive benefits after hearing deprivation by enhancing non-auditory skills, such as superior visual speech reading skills (Rouger et al., 2007). On the other hand, it has also been shown to be related to behavioral measures of speech performance (Strelnikov et al., 2013; Anderson et al., 2017; Fullerton et al., 2023). This adult cochlear implant research literature suggests that cross-modal plasticity may be another factor affecting speech perception outcomes in children with cochlear implants. However, the effects of cochlear implantation on the cross-modal plasticity of the auditory visual cortex in children with hearing loss have not yet been fully defined in clinic, and the mechanism of cochlear implantation affecting the recovery of hearing and speech function needs to be further explored. Therefore, this study aims to analyze this and provide a basis for clinical intervention, as reported below.

## 2 Materials and methods

### 2.1 General demographic profile

The prelingually deaf group consisted of 20 children admitted to our hospital between February 2020 and February 2022, who underwent cochlear implantation and speech rehabilitation training. Additionally, a control group of 20 healthy children, who underwent physical examinations at our hospital during the same timeframe, was chosen. Inclusion criteria encompassed: (1) age between 2 and 7 years old; (2) prelingually deaf children with cochlear implants; (3) ability to cooperate with cortical activity detection, auditory, and visual function assessments; (4) normal hearing function in healthy children as indicated by standard auditory tests; (5) native Mandarin speakers; (6) informed consent obtained from parents or guardians. Exclusion criteria comprised: (1) acute inflammatory infections, acute/chronic renal failure, lung disease, immune disorders, coronary artery disease, chronic liver disease, or mental disorders; (2) abnormal cochlear electrode placement or cochlear abnormalities; (3) unwillingness to cooperate with examinations.

This research protocol has been approved by the ethics committee.

### 2.2 Detection and evaluation methods

The control group of children underwent assessment solely on the day of their physical examination. In contrast, the group diagnosed with prelingual deafness underwent evaluation prior to surgery, followed by assessments during the first and second weeks post-surgery, and subsequently at intervals of 1, 3, 6, 9, and 12 months post-surgery.

#### 2.2.1 Brain cortical activity detection

The EGS400 electroencephalogram (EEG) system that caters to both pediatric and adult populations was manufactured by EGI, a US-based company, and was utilized for detection purposes. Prior to conducting examinations, the child and their guardians were provided with detailed instructions by the examiner. During testing, patients were comfortably seated while wearing an electrode cap.

1. Visual Stimulation Test: Each patient underwent an evaluation process when event-related potential (ERP) was triggered through visual stimulation tests. These tests employed silent or auditory pictures controlled by specialized psychological experiment software called Eprime 2.0. The pictures were displayed on a high-quality video graphic array computer screen whose dimensions were 350 × 350 pixels. Both types of pictures constituted independent stimulus sequences, with each stimulus lasting 1,000 ms. A total of 60 stimuli were presented at intervals ranging between 1,700 and 1,900 ms. Within each sequence, one deviant stimulus, differed from previous ones, was introduced after every 5 to 10 stimuli. Following the completion of each picture stimulation session, patients were given approximately 10–15 s of rest.
2. Data Processing: After collecting data using Brainstorm, each patient’s averaged ERP segments were generated using an ERP analysis software. These averaged data sets facilitated calculations pertaining to brain functional activity source localization. This process enabled the recording of changes in auditory and visual cortical activities through electromagnetic intensity values. In the calculation process, the average models of finite difference model (FDM), computed tomography (CT) and fMRI were used to reconstruct and determine the distribution of 2,447 dipoles in the cerebral cortex. Standardized lower solution brain electromagnetic tomography was used for the inverse method. In the inverse model, regularization levels are adjusted to stabilize the source location results. The source location results showed the electromagnetic intensity signal value, which was used to represent the cerebral cortex activity level and recorded the changes of auditory and visual cortex activity (electromagnetic intensity value) of each subject.

#### 2.2.2 Assessment of auditory and language function recovery

The assessment tools utilized in this study to evaluate the recovery of auditory and speech functions include the Categories of Auditory Performance (CAP) questionnaire and the Speech Intelligibility Rate (SIR) questionnaire. The CAP assessment, as outlined by experts, delineates various levels of auditory performance through 0 to 9 (Albalawi et al., 2019). For this study, corresponding scores were allocated for each level, with higher CAP scores indicative of superior auditory behavior. Similarly, the SIR assessment establishes distinct levels of speech intelligibility (Wang et al., 2013). The SIR score corresponds directly to language proficiency, with a higher score denoting superior proficiency.

### 2.3 Statistical methods

The data analysis utilized SPSS 20.0 software. Count data were expressed as frequencies (percentages) [*n* (%)] and subjected to chi-square testing. For normally distributed continuous variables, descriptive statistics were reported as mean ± standard deviation ( $\bar{x}$ ± s), with significance determined using t-tests. Pearson’s linear correlation analysis was employed to evaluate the relationship between auditory and visual cortical activities in pediatric patients with CAP and SIR scores. Statistical significance was set at *p* < 0.05.

## 3 Results

### 3.1 Comparison of auditory cortical activity between two groups

Since the control group consisted of typically developing children, only pertinent assessments were administered on the day of their physical examination to contrast their outcomes with those of the prelingually deaf group across various time periods. Notably, the auditory cortical electromagnetic intensity values in the prelingually deaf group showed a statistically significant rise compared to those in the control group from pre-surgery through 9 months post-surgery (*p* < 0.05).

However, there was no notable distinction in auditory cortical electromagnetic intensity values between the two groups at the 12-month mark post-surgery (*p* > 0.05). Within the prelingually deaf group, a gradual increase in auditory cortical electromagnetic intensity values was observed initially, from pre-surgery to 1 month post-surgery, followed by a subsequent gradual decline beginning at 3 months post-surgery, eventually stabilizing to a level akin to that observed in the control group at the 12-month post-surgery assessment (Table 1).

**Table 1:** Comparative analysis of auditory cortical electromagnetic intensity between the two groups (nA.m, *x*¯ ± *s*).

Groups	Pre-surgery	1 week post-surgery	2 weeks post-surgery	1 month post-surgery	3 months post-surgery	6 months post-surgery	9 months post-surgery	12 months post-surgery
Prelingual deaf group (*n* = 20)	1.39 ± 0.20	1.41 ± 0.18	1.43 ± 0.13	1.59 ± 0.16^abc^	1.50 ± 0.10^ad^	1.34 ± 0.18^de^	1.30 ± 0.17^cde^	1.07 ± 0.21^abcdefg^
Control group (*n* = 20)	1.08 ± 0.24	1.08 ± 0.24	1.08 ± 0.24	1.08 ± 0.24	1.08 ± 0.24	1.08 ± 0.24	1.08 ± 0.24	1.08 ± 0.24
*t*	4.438	4.919	5.735	7.907	7.224	3.876	3.345	0.140
*p*	<0.001	<0.001	<0.001	<0.001	<0.001	<0.001	0.002	0.889

Compared with pre-surgery, ^a^*p* < 0.05; Compared with 1 week post-surgery, ^b^*p* < 0.05; Compared with 2 weeks post-surgery, ^c^*p* < 0.05; Compared with 1 month post-surgery, ^d^*p* < 0.05; Compared with 3 months post-surgery, ^e^*p* < 0.05; Compared with 6 months post-surgery, ^f^*p* < 0.05; Compared with 9 months post-surgery, ^g^*p* < 0.05.

### 3.2 Comparison of visual cortical activity between two groups

The prelingually deaf group demonstrated a notable rise in visual cortical electromagnetic intensity values compared to the control group from pre-surgery through 9 months post-surgery, as evidenced by statistically significant differences (*p* < 0.05). However, by the 12-month mark post-surgery, there was no significant variance in visual cortical electromagnetic intensity values between the two groups (*p* > 0.05). Specifically, within the prelingually deaf group, the peak in visual cortical electromagnetic intensity occurred at 1 month post-surgery, followed by a gradual decline from 3 months post-surgery onwards, eventually stabilizing near levels akin to those observed in the control group by the 12-month post-surgery mark (Table 2).

**Table 2:** Comparative analysis of visual cortical electromagnetic intensity between the two groups (nA.m, *x*¯ ± *s*).

Groups	Pre-surgery	1 week post-surgery	2 weeks post-surgery	1 month post-surgery	3 months post-surgery	6 months post-surgery	9 months post-surgery	12 months post-surgery
Prelingual deaf group (*n* = 20)	2.18 ± 0.24	2.19 ± 0.16	2.21 ± 0.20	2.40 ± 0.18^abc^	2.29 ± 0.20^c^	2.08 ± 0.15^abcde^	1.75 ± 0.16^abcdef^	1.67 ± 0.22^abcdef^
Control group (*n* = 20)	1.62 ± 0.18	1.62 ± 0.18	1.62 ± 0.18	1.62 ± 0.18	1.62 ± 0.18	1.62 ± 0.18	1.62 ± 0.18	1.62 ± 0.18
*t*	8.348	10.585	9.806	13.703	11.136	8.780	2.414	0.787
*p*	<0.001	<0.001	<0.001	<0.001	<0.001	<0.001	0.021	0.436

Compared with pre-surgery, ^a^*p* < 0.05; Compared with 1 week post-surgery, ^b^*p* < 0.05; Compared with 2 weeks post-surgery, ^c^*p* < 0.05; Compared with 1 month post-surgery, ^d^*p* < 0.05; Compared with 3 months post-surgery, ^e^*p* < 0.05; Compared with 6 months post-surgery, ^f^*p* < 0.05.

### 3.3 Comparison of CAP scores between two groups

The prelingually deaf group demonstrated markedly lower CAP scores compared to the control group, both preoperatively and at the 12-month follow-up (*p* < 0.05). However, postoperative CAP scores in the prelingually deaf group displayed a progressive augmentation from 1 week to 12 months post-surgery, exceeding their preoperative levels (*p* < 0.05, Table 3).

**Table 3:** Comparative analysis of CAP scores between the two groups (point, *x*¯ ± *s*).

Groups	Pre-surgery	1 week post-surgery	2 weeks post-surgery	1 month post-surgery	3 months post-surgery	6 months post-surgery	9 months post-surgery	12 months post-surgery
Prelingual deaf group (*n* = 20)	0.86 ± 0.18	2.30 ± 0.12^a^	2.30 ± 0.18^a^	2.88 ± 0.23^abc^	3.14 ± 0.21^abcd^	4.52 ± 1.16^abcde^	5.80 ± 1.57^abcdef^	6.81 ± 1.69^abcde^
Control group (*n* = 20)	8.10 ± 0.24	8.10 ± 0.24	8.10 ± 0.24	8.10 ± 0.24	8.10 ± 0.24	8.10 ± 0.24	8.10 ± 0.24	8.10 ± 0.24
*t*	107.928	96.667	86.461	70.227	69.556	13.516	6.476	3.380
*p*	<0.001	<0.001	<0.001	<0.001	<0.001	<0.001	<0.001	0.002

Compared with pre-surgery, ^a^*p* < 0.05; Compared with 1 week post-surgery, ^b^*p* < 0.05; Compared with 2 weeks post-surgery, ^c^*p* < 0.05; Compared with 1 month post-surgery, ^d^*p* < 0.05; Compared with 3 months post-surgery, ^e^*p* < 0.05; Compared with 6 months post-surgery, ^f^*p* < 0.05.

### 3.4 Comparison of SIR scores between two groups

Before surgery and during the 12-month follow-up, the prelingually deaf group exhibited significantly lower SIR scores compared to the control group (*p* < 0.05). There was some noteworthy improvement in postoperative SIR scores in the prelingually deaf group from 1 week to 12 months after surgery (*p* < 0.05, Table 4).

**Table 4:** Comparative analysis of SIR scores between the two groups (point, *x*¯ ± *s*).

Groups	Pre-surgery	1 week post-surgery	2 weeks post-surgery	1 month post-surgery	3 months post-surgery	6 months post-surgery	9 months post-surgery	12 months post-surgery
Prelingual deaf group (*n* = 20)	0.55 ± 0.10	2.03 ± 0.19^a^	2.06 ± 0.21^a^	2.37 ± 0.19^abc^	2.61 ± 0.25^abcd^	2.93 ± 0.39^abcde^	3.18 ± 0.41^abcde^	3.45 ± 0.72^abcdef^
Control group (*n* = 20)	4.80 ± 0.12	4.80 ± 0.12	4.80 ± 0.12	4.80 ± 0.12	4.80 ± 0.12	4.80 ± 0.12	4.80 ± 0.12	4.80 ± 0.12
*t*	121.677	55.125	50.663	48.359	35.318	20.495	16.959	8.271
*p*	<0.001	<0.001	<0.001	<0.001	<0.001	<0.001	<0.001	<0.001

Compared with pre-surgery, ^a^*p* < 0.05; Compared with 1 week post-surgery, ^b^*p* < 0.05; Compared with 2 weeks post-surgery, ^c^*p* < 0.05; Compared with 1 month post-surgery, ^d^*p* < 0.05; Compared with 3 months post-surgery, ^e^*p* < 0.05; Compared with 6 months post-surgery, ^f^*p* < 0.05.

### 3.5 Correlation analysis between auditory–visual cortical activity in prelingually deaf children and CAP/SIR scores

Significant fluctuations in auditory–visual cortical activity were noted among prelingually deaf children, with activity levels approaching those of the control group around 12 months post-surgery. Consequently, this study primarily investigates correlations among different indicators during this specific time frame. The results reveal a negative correlation between electromagnetic intensity values of auditory–visual cortical activity and CAP/SIR scores in these patients (*p* < 0.05, Table 5).

**Table 5:** Correlation analysis of auditory–visual cortical activity and CAP/SIR Scores in prelingually deaf children.

Index	CAP score		SIR score	
	*r*	*p*	*r*	*p*
Auditory cortical electromagnetic intensity values	−0.442	<0.001	−0.511	<0.001
Visual cortical electromagnetic intensity values	−0.428	<0.001	−0.489	<0.001

## 4 Discussion

Childhood deafness exerts a profound impact on both language and auditory development, posing significant challenges for affected families and society at large (Zheng, 2019). From recent data, it can be seen that there is a sizable population of approximately 427,000 disabled children aged 0–14 in China, the majority of whom grapple with severe disabilities necessitating early intervention strategies (Meng and Zhang, 2021). Among the interventions, cochlear implantation stands out as a widely adopted approach for individuals with hearing impairments, necessitating postoperative speech rehabilitation to bolster vocabulary acquisition and facilitate the recuperation of hearing and speech capabilities (Alzhrani et al., 2021; Aljazeeri et al., 2022). Yet, the precise mechanisms through which cochlear implantation fosters the restoration of auditory and speech functions in prelingually deaf children, who have experienced hearing loss before developing language skills, remain elusive.

It is becoming evident from emerging research that during the initial phases of cochlear implantation, the auditory cortex undergoes adaptations in visual processing, indicating potential alterations in brain activity across auditory and visual cortices among patients (Zhao et al., 2020). Given the pivotal importance of the first-year post-implantation for individuals with prelingual deafness in terms of hearing and speech rehabilitation, this study aims to examine fluctuations in cortical activity, alongside changes in auditory and speech functions during this critical period.

The primary objective of the study is to explore potential associations between cortical activity patterns and the restoration of auditory and speech functions in these patients, with the overarching goal of advancing our understanding of the underlying mechanisms. The results of this study indicate a notable increase in auditory and visual cortical electromagnetic intensity values in the prelingually deaf group following cochlear implant surgery, compared to the control group.

This increase peaked at 1 month post-surgery, with subsequent gradual normalization observed over the following months. These findings suggest the presence of abnormal activity in both auditory and visual cortices during the early stages of cochlear implantation in children with hearing loss. However, this aberrant activity diminishes over time, indicating a reversal of cross-modal functional reorganization within the brain. This phenomenon may arise from the initial inability of the auditory mechanisms of these children to compensate for the original visual system when utilizing cochlear implants, resulting in abnormal cortical activity during this period.

Nevertheless, usage of cochlear implant for more than a month prompts rapid development and heightened plasticity in the auditory cortex, leading to a reversal of cross-modal functional reorganization and adjustments in both auditory and visual cortical activities. Furthermore, prolonged speech training facilitates gradual normalization in both auditory and visual cortical activities.

The findings indicate that the visual cortex does not play a direct role in compensatory mechanisms. Thus, post-cochlear implantation, auditory input may shift central functional control away from vision, leading to a gradual decrease in visual cortical activity and promoting cross-modal functional reorganization in the brain (Xin et al., 2022). Consequently, prelingually deaf children initially display abnormal cortical activity after cochlear implantation, followed by a reversal phenomenon and a gradual return to normalcy.

This investigation uncovered a positive correlation between the duration of cochlear implantation in pediatric patients and the degree of improvement in auditory and speech functions, consistent with prior studies (Wang et al., 2022; Zhou et al., 2022). Subsequent examination of the interplay between cortical activity in children’s brains and the restoration of auditory and speech functions revealed a strong relationship between electromagnetic intensity values within auditory and visual cortices, alongside CAP and SIR scores. This underscores the critical role of cortical activity in fostering auditory and visual recovery.

Some scholars suggest that overreliance on visual aids post-cochlear implantation could impede optimal auditory function recovery. The restructuring of postoperative auditory cortex activity is pivotal in promoting auditory restoration, highlighting the impact of auditory cortical activity on auditory function (Sun et al., 2021). The reversal and restructuring of cross-modal functions in the cerebral cortex following cochlear implantation surgery are associated with the recovery of auditory and speech functions in deaf patients.

The potential normalization of abnormal activities in the auditory and visual cortices post-cochlear implantation may constitute one of the mechanisms influencing the efficacy of hearing and speech recovery. Despite previous reports on the effects of cochlear implantation on hearing, the underlying mechanisms remain elusive. This study brings forth potential mechanisms to offer evidence-based support for clinical interventions.

## 5 Conclusion

The timely implementation of cochlear implants in prelingually deaf children leads to distinctive auditory–visual cortical patterns. Over time, prolonged implantation triggers a reversal of cross-modal functional changes in the brain, gradually restoring typical activity. This phenomenon plays a crucial role in the restoration of auditory and speech capabilities in affected individuals. However, it is crucial to recognize the limitations of this study, including the possibility of subjective bias in scale assessments. Thus, there is a pressing need for more dependable methodologies to assess progress towards recovery.

## Data Availability

The original contributions presented in the study are included in the article/supplementary material, further inquiries can be directed to the corresponding author.
